# Regional and inter-regional economic rules and the enforcement of the right to health: The case of Colombia

**DOI:** 10.1177/1468018115599821

**Published:** 2015-12

**Authors:** Liliana Lizarazo Rodríguez, Philippe De Lombaerde

**Affiliations:** Lawyer and Independent Consultant, Belgium; United Nations University (UNU-CRIS), Belgium

**Keywords:** Andean Community, Colombia, free trade agreements, regional integration, right to health

## Abstract

The regional policy level is often seen as a (potential) source of progressive policy-making in health (and in social policy more widely), complementing or substituting national policy levels, which are perceived as underperforming. While it can certainly be argued that there are important opportunities to adopt regional approaches to tackle border-crossing health issues, this article draws the attention to the fact that the linkage between (inter-)regional and national policy levels is not uni-directional. While in some instances the regional level may indeed take the lead in the promotion of (the right to) health, in other instances it may well be the other way round. This article focuses on the case of Colombia, where international economic rules have deeply permeated public policies in the health sector. On one hand, Colombia has been opening markets through the conclusion of regional integration arrangements (e.g. Andean Community and the Pacific Alliance) and the new generation of Free Trade Agreements. On the other hand, Colombia has been one of the most active emerging countries in promoting the right to health as a justiciable fundamental right, in line with the International Covenant on Economic, Social, and Cultural Rights of the United Nations mainly due to the judicial activism of the Colombian Constitutional Court with interesting implications for regional social governance. The article shows that national courts can play an important role in the protection of the right to health in a context of economic integration and the absence of regional balancing policies.

## Introduction

The potential for the design and implementation of regional social policies in general (Deacon et al., 2010; Kaasch and Stubbs, 2014), and for new regional health policy initiatives in particular (Lee and Pang, 2015), is often argued. The regional level is thereby seen as a potential source of progressive policy-making, complementing or substituting national levels, which are perceived as underperforming. This is very much in line with the orientation of the broader literature (and normative theorizing) on global health governance (Lee and Kamradt-Scott, 2014; McInnes et al., 2012; McInnes and Lee, 2012). While we recognize that especially in cases of border-crossing health issues (communicable diseases, migration-related health issues, etc.) regional health policies are to be recommended and that outward regional health diplomacy can contribute crucially to defending the interests of the region in a context of globalization, we argue that the linkage between international/inter-regional/regional and national policy levels is not uni-directional. While in some instances the regional level may indeed take the lead in the promotion of (the right to) health, in other instances it may well be the other way round.

In this article, the latter instance will be more closely looked at in the case of Colombia. We show that while there is a potential for regions to promote health, in practice, regional and inter-regional economic rules do not necessarily work in that direction so that national institutions (mostly courts), or other actors, have to step in and protect the right to health in the absence of regional balancing policies.

The article speaks therefore to the more critical approaches to the contribution of regional arrangements, especially the ones in the economic sphere, to the protection of social rights, as exemplified by O’Brien (2008) in the area of labor rights. The question which emerges then is whether the literature on regional health policy or governance does not need more balanced assessments by taking overlapping memberships (e.g. the Union of South American Nations [UNASUR], Andean Community [CAN], and free trade agreements [FTAs]) into account as well as interacting policy areas (e.g. health and trade).

The article speaks also to the literature on health diplomacy and the protection of the right to health. In effect, this literature emphasizes the role that, for example, regional organizations and civil society actors can play as intermediaries in the relationships between national health policymakers, health service providers, and the people in need of medical services (or, rights bearers).^1^ Our contribution will be to show that at least in the case of Colombia, the role played by regional organizations (through health diplomacy or other actions) should not be overestimated, while national actors such as courts (and the justiciability of the right to health) are playing an important role in the policy game. Outside the legal discipline, this role is somewhat underestimated or even ignored.

The case of Colombia is relevant for several reasons. First, the protection of constitutional rights by its Constitutional Court is internationally recognized (Bonilla, 2014; ICJ, 2008; Landau, 2014; Rodríguez, 2011; Uprimny, 2006). Second, in times where simplistic ideas abound on bipolar politics (i.e. left *vs* right) in South America, the case illustrates that social policy orientation in the region should be nuanced. And third, Colombia is a typical case of a country where policy-making takes place in an increasingly complex environment, characterized by multiple international commitments, including with the World Bank, International Monetary Fund (IMF), the World Trade Organization (WTO), the United Nations (UN), the CAN, the UNASUR, the Pacific Alliance, and a series of FTAs.

The article is organized as follows: In section ‘Contextualizing the protection of the right to health’, the protection of the right to health will first be contextualized by referring to the international regulatory context. This is followed by a discussion of how the right to health is protected in Colombia, with specific attention for its constitutional framework and court activism (section ‘The right to health in Colombia’). In section ‘(Inter-)Regional Integration and the Right to Health’, we analyze how the protection of the right comes under pressure in a context of regional and inter-regional economic integration arrangements (CAN and recent FTAs), and how the protection of the right to health is balanced (or not) with the protection of Intellectual Property Rights (IPR). Section ‘Conclusion’ concludes.

## Contextualizing the protection of the right to health

The concept of the international human right to health is a framework for states seeking to promote health care, in accordance with their economic resources and cultural mores (Kinney, 2001: 1458). The most important international treaty recognizing the right to health is the Universal Declaration of Human Rights (UDHR) and the International Covenant on Economic, Social, and Cultural Rights (ICESCR). The UN also created specialized institutions such as the World Health Organization (WHO). At the regional level, the Organization of American States (OAS) recognized health as a public good in the San Salvador Pact (Kinney, 2001: 1460–1462).

General Comment (GC) 14 of the ICESCR Committee is the most important parameter defining the scope of the international right to health. It requires national health systems to be institutionally capable to realize this right, which includes *the availability, accessibility, acceptability, and quality of needed health care services and facilities*. It also defines the obligations of the states as the duties of respect, protection, and fulfillment. In addition, the violation of the right to health was defined as the unwillingness *to use the maximum of its available resources for* [its] *realization*. Concerning the duties of fulfillment, failures in recognizing the right to health mainly by legislative way, complemented with policies focused toward vulnerable groups, were seen as concrete violations (Kinney, 2001: 1467–1471). The enforcement of the right depends on the concrete economic and cultural framework of each country, but the promotion of *universal outcome measures* seeks to evaluate its implementation with the help of human development indicators to monitor the compliance of state duties (Kinney, 2001: 1472–1474).

At the national level, a general trend has been the progressive constitutionalization of the right to health. Kinney and Clark (2004: 290) found that, first, the main constitutional provisions worldwide aim at a healthy environment and workplace, the protection of the right to life, and to promote general welfare. Second, 67.5% of the constitutions worldwide include provisions addressing health care. However, many countries that spend important resources on health do not have relevant constitutional rules regarding this right. In countries with the highest *per capita* government expenditures for health care, only 3 out of 16 have constitutional health duties. Third, no correlation was found between the intensity of constitutional commitments and the government expenditures for health. The importance of legal remedies as access to courts and the opportunity to challenge policymakers who fail to enforce constitutional mandates also varies across countries. They conclude that legal actions are not the best option to obtain health care because when judges adjudicate in this sense, they become policymakers in an environment of scarcity. As a result, policy duties created by the constitution and international human rights treaties are standards to evaluate the performance of governments in the realization of the Economic, Social, and Cultural Rights (ESCR). Court intervention by providing *solutions* for governmental failures in the enforcement of rights is limited by budgetary restrictions which determine the realization of a satisfactory level of health care (Kinney and Clark, 2004: 291, 296, 300–301).

According to the international literature on the right to health, the role played by the regional level is not very visible, and instead the duties of the countries in this respect are gradually expanding and becoming more demanding.

## The right to health in Colombia

Provided that the ESCR have the status of constitutional rights in Colombia, the Court has been transforming their nature and scope by considering them as fundamental rights, that is, the State should guarantee them in an immediate way, in strong contrast with the traditional approach that rather considers them as goals of the State whose obligations are of progressive compliance. ESCR have been qualified as the corner stone of the legal reasoning of the Court, and among them, the right to health represents the paradigm of judicial protection of ESCR (Cepeda, 2004: 617).

Following the general understandings at the international level, the Court also admitted that those rights have an *essential, non-negotiable nucleus that may not be restricted* and another part of progressive fulfillment defined by law, that is, ESCR become fundamental when disregarding them may affect the dignity of a person and should be fulfilled immediately, including by judicial means (Cepeda, 2004: 617–642).

The Court argued that if the State does not allocate fiscal resources to supply basic needs, the judicial power can intervene because the protection of ESCR is one of its main functions in view of reaching the objectives of the Legal Social State (Cepeda, 2004). This position of the Court has been questioned by economic authorities who consider the fulfillment of the ESCR as a goal of the State so that its duties can be limited, even if the judiciary orders the provision of the corresponding health services.

In 2010, the United Nations Economic and Social Council [UNESC] evaluated the progress of Colombia on public health policy and highlighted the lack of updated information and statistics to assess whether ESCR were being implemented by Colombia. The Council recommended the strengthening of public health policy, to ensure universal access to healthcare services, especially for vulnerable groups (United Nations Economic and Social Council [UNESC], 2010: 25). This position is the one shared by the Court which, despite economic constraints and criticisms, seems to be implementing binding rules in line with the international guidelines of the ICESCR.^2^ Although the impact of constitutional case law on the enforcement of the right to health is remarkable, universal coverage of good quality is not yet being granted (López, 2009: 410-11; Procuraduría General de la Nación, 2008: 171, 179). The Human Rights Council (HRC, 2013) of the UN highlighted the unification of the healthcare systems in 2012 promoted by the Court, which means that the benefits are now equal for the contributory regime and the subsidized health plan, with a health insurance coverage of 96% (p. 41).

This unification of the healthcare system has been reached because of the action of the Court. In fact, ruling T860/07 is considered as a real *judicial statute* on the provision of health services in which the Court ordered the government to take measures to eliminate the *regulation failures* of health programs and, to assure its integral updating, the unification of both regimes and the appropriate and efficient provision of the service, emphasizing that the goal of universal coverage is to be reached.

This ruling is a real landmark case for the judicialization of health policies and it is the consequence of the great impact of health litigation on health policies in Colombia. Although the case of Colombia is not unique (ICJ, 2008: 65–69), a study put in perspective the relevance of this case by showing that Colombia is the middle-income country *with the highest per capita rate of right to health litigation* with 3289 legal actions per million of persons, followed by Brazil (206), Costa Rica (109), Argentina (29), South Africa (0.3), and India (0.2) (Mæstad et al., 2011, quoted by Lamprea, 2013: 22).

According to these ‘jurisprudencial guidelines’ for health policies, human dignity gives a fundamental character to the right to health, and therefore, human dignity may condition healthcare services.^3^ This means that the duty of implementation of public health policy should be harmonized with guaranteeing the effectiveness of constitutional rights.

A relevant aspect of public health policy is the regulation of the pharmaceutical market which has a direct relation with regional integration and which finds – in Colombia – binding parameters in the law but also in related case law. The cost of medicines is indeed one of the major concerns in developing countries’ healthcare systems. In Colombia, in the period 2006–2008, public expenditure on pharmaceuticals represented 1.3% of the gross domestic product (GDP), increasing to 3.2% of GDP in 2009 (Andia, 2011; quoted by Lamprea, 2013: 28), a situation which was mainly explained by the deregulation of pharmaceutical prices by the government in 2006,^4^ which together with the widespread use of health litigation provoked a strong pressure on the financing of health services (Lamprea, 2013: 28–32).^5^ In 2012, the government started with a policy of price control of medicines,^6^ in line with the order of the Court^7^ to the government to solve the critical situation of overruns of prices of medicines for the health system. The first measures regulated the price of more than 300 medicines, taking as a reference a basket of international prices and unified the prices of medicines for re-payment purposes by the public funds.^8^

The Court elevated the level of regulation of the market of medicines to a statutory law because its functioning is a determining factor to guarantee the three essential elements of the right to health: availability, accessibility, and quality.^9^ The Court supported the price control of medicines by the government because a strict regulation of the market of medicines would mitigate unsuitable practices in the health system, which make that the prices of medicines are higher than in other countries or than international reference values. In short, in Colombia, the accessibility of medicines is now a constitutional duty, that is, pharmaceutical enterprises have also a constitutional social function when deploying their activities, which implies duties, and the way to reach it is by the regulation of the prices of medicines in all phases of production and commercialization until the final users of the product.

As a result, the Colombian government is also advancing new regulations for biological medicines whose patents will soon expire, and therefore, their price control is also an issue linked to the concept of bio-similar medicines^10^ whose relevance is high because biological medicines represent almost 30% of the pharmaceutical market in Colombia and they are mostly financed by the public health system (Minsalud, 2014).

The development of bio-generics is a more complex issue than the chemical generics. The issue concerns price controls, patents, but above all the technological capacity of emerging countries to regulate these processes and to produce biosimilars. The regulation of biosimilars by emerging economies has been strongly opposed by the United States and the European Union (EU) and by the Biotechnology Industry Organization (BIO) arguing concerns about public health.^11^ Even more, they are announcing a potential consultation before the WTO for those regulations seen as technical barriers to trade.^12^ In Latin America, Brazil and Cuba are leading the way on this issue, but it is a field where regional cooperation would appear as essential to reach a good level of regulation and production. The UNASUR is quoted as an adequate forum to reach concrete goals in this respect (Feijó et al., 2012, 2014; Lage, 2011).

## (Inter-)regional integration and the right to health

Regional integration processes have gradually covered other domains than trade, the EU being the most emblematic case. In Latin America, which is characterized by multiple regional schemes, it is the UNASUR which seems to privilege political concerns over commercial issues and which has entered the health policy area. Concerning the right to health, the main tension between its enforcement and international trade commitments of many regional and bilateral agreements is related to reaching universal coverage in health care in the presence of strong measures to protect IPR (Crook, 2005: 538; Van Hees, 2004).

According to GC14, access to medicines is a crucial component of the right to health, which in turn is linked to the right to life (Ely, 2003). The Doha Declaration on the Agreement on Trade-Related Aspects of Intellectual Property Rights (TRIPS) and Public Health of 2001^13^ sought to flexibilize TRIPS enforcement in cases of public health crises (access to medicines) in developing countries. This declaration sought to balance incentives for the production of IPR (pharmaceutical subsidies or public funding to promote innovation) with the promotion of its widespread use (flexibilization of patent regulations in developing countries, for example, by issuing compulsory licenses or by importing generic medicines) (Crook, 2005: 545, 550).

In the ‘Special 301’ Report,^14^ the US Trade Representative (USTR) also refers to this conflictive relation between IPR and the right to health. The United States recognizes, in line with the Doha Declaration, the right of trading partners to protect public health (especially the generalized access to medicines) but without forgetting that the patent system is crucial for the *creation of new and innovative lifesaving medicines* (USTR, [2013] 2014: 22). It added that the United States respects the right of trading partners to grant compulsory licenses but on the condition that IPR systems remain respected. In other words, the United States seeks a balancing between the need of granting *compulsory licenses to export pharmaceutical products to countries that cannot produce drugs for themselves* but seeking to *reduce market access barriers that US pharmaceutical and medical device companies face in many countries* (USTR, [2013] 2014: 23).

The emergence of the new generation of FTAs makes the issue more complex because these agreements are considered as TRIPS-plus with respect to the protection of IPR. That is, the United States and the EU seek to go further in the protection of IPR of their pharmaceutical enterprises. However, a study of UNDP-UNAIDS (2012) concluded that the advantages of TRIPS-plus protection in the new generation FTAs, that is, that they increase foreign investment and/or innovation, have not been fully demonstrated. On the contrary, the study alerted about their possible detrimental effects worldwide.^15^

In Latin America, and in the particular case of Colombia, regional/bilateral integration has been closely related with trade and investment and therefore CAN regulations have been modified in order to allow the signature of FTAs by their member states. In contrast, UNASUR has been trying to propose regional measures seeking to protect the right to health at the regional level, for example, by the proposal to create the *Banco de Precios de Medicamentos*, but these proposals still lack implementation. New regional integration groups such as the Pacific Alliance are again more focalized on commerce than the UNASUR, and members of the Alliance have been more active in the promotion of FTAs than the other members of the UNASUR. This explains why the Alliance does not have any measure concerning health issues, but on the contrary, they seek to strengthen the IPR regimes of its member states. However, Colombia made some reservations to the treaty with respect to its regulatory competences in the area of social services such as health.^16^

At the level of the FTAs, the case of Colombia illustrates how the judiciary is trying to respect ICESCR and the guidelines of the Committee, but, at the same time, it does not oppose public policies aligned with trade liberalization. Although defenders of FTAs argue that the new generation FTAs include some conditionality related to the duty to respect human rights in general (HRC, 2013: 94), this view contrasts with the concluding remarks of the UNESC Council (UNESC, 2010: 10–11), which put in evidence the potential negative effects of the FTAs, by referring concretely to the COL-US FTA provisions on IPR, because it might increase the price of medicines and affect the right to health. For this reason, the UNESC Council urged Colombia to take measures to ensure the compliance with ESCR duties in all FTAs as well as to enforce policies to protect these rights against the negative impact of the agreements, thereby concretely referring to the price of medicines.

The next sections further analyze how the new generation FTAs have clearly played a role in the scope and accessibility to health services and how some regional organizations, despite their good intentions, have also been subordinated to the binding rules of FTAs concerning the protection and enlargement of IPR at the cost of the right to health.

### Andean Community (CAN)

As early as 1971, the Andean Health Organism (ORAS-CONHU)^17^ was created as an intergovernmental institution, seeking to develop coordinated actions to grant the right to health. In 1999, the Andean Social Charter sought to find a compromise among member states on the universalization of all rights, including ESCR. The charter also promoted the non-privatization of the basic health services, the use of traditional medicine, and international cooperation in the prioritization of health programs. In 2012, the Andean Parliament sought to give it binding character, but until now it remains a declaration of intentions.

CAN member states have also been working with Mercosur and the Amazonian Cooperation Treaty Organization (OTCA)^18^ to develop a South American agenda^19^ which is mainly being conducted by the UNASUR.^20^ The scope and impact of these forms of regional cooperation are not clear (Ortíz et al., 2011). So far, concrete common health policies of the UNASUR have not necessarily materialized and are not necessarily very visible at the level of the member states.

CAN, by contrast, is being recognized as a pioneer regional integration group in regulating IPR issues at the regional level. However, it has institutionally been affected by the signature of FTAs by Colombia and Peru, countries that promoted prior reforms required for this purpose (Helfer and Alter, 2014). The communitarian IPR regime has therefore been adapted to the parameters of the United States and the EU FTAs, which constitutes a threat to the enforcement of the right to health to the benefit of IPR for trade partners.

The first step in the institutional change was taken when Decision 598/2004 revoked Decision 322/93 to allow FTA negotiations by member states with third countries. Then, the reforms to the Andean IPR regime were actively promoted by Colombia and Peru because the Andean Tribunal of Justice (ATJ) had clearly ruled that CAN law is binding for member states, including for international treaties signed by them. The ATJ held that patent registration of proof data for medicines would threaten free competition but also the right to access to medicines because it may be a way to extending patents (Ruling 114-AI-2004; quoted by Uribe, 2007: 115). As a result, Decision 632/06 interpreted Decision 486/00^21^ and left each member state the choice of whether to protect proof data, including by preventing third party commercialization based on proof data information. This reform sought to validate a regulation of the Colombian government concerning the exclusive protection of test data rejected by the ATJ (Helfer and Alter, 2014). In fact, the protection of proof data has been incorporated into the FTAs that Colombia signed with the United States (16) and with the EU (231), a rule that has been seen as an obstacle for the production of generic medicines (Wolfram, 2011, quoted by Saura Estapà, 2013).

Second, Decision 689/08 modified Decision 486/00, a reform strongly opposed by Bolivia which challenged the new decision before the ATJ.^22^ This reform was proposed by Peru to facilitate FTA negotiations with the United States and sought to allow member states to develop some IPR through national legislation, taking into account socioeconomic asymmetries and multilateral standards.^23^

In short, despite the institutional crisis of the CAN, the binding character of its IPR regulations is not a matter of discussion,^24^ and national powers are due to apply it, including the judicial branch. Preliminary rulings are often used by national tribunals of member states, and the rulings of the ATJ are part of the legal system. Helfer and Alter (2014: 247–248) found that in 2007, IPR preliminary rulings of the ATJ represented around 97% of their total preliminary rulings and Colombia is recognized as the most active member of the Andean Community in IPR protection. In fact, it is the most active member state in referring cases to the ATJ (around 2/3 of the ATJ preliminary rulings on IPR).^25^ Helfer and Alter (2014) found that intellectual property (IP) administrative agencies supported by the ATJ have tried to balance IPR in favor of access to medicines, but with the pressures of the TRIPS and the Colombian and Peruvian FTAs, this position is being weakened at the cost of public health in these countries.

### New generation FTAs and the right to health

Tensions between FTAs signed by Colombia with both the United States (2006) and the EU (2012), on one hand, and the protection of the right to health, on the other, have mainly been related with higher protection of IPR vis-a-vis TRIPS.^26^ Although this should be a policy discussion, in Colombia the judiciary has been implicated in the analysis of these issues, giving in general a higher rank to the right to health than IPR by means of the constitutional control of statutes approving the FTAs by the Congress.^27^

The Court upheld all recently signed FTAs but clarifying that the authorities in charge of the execution and enforcement of FTAs are constitutionally bound and that their acts are subject to administrative and/or judicial control. This means that the effective constitutionality control should be realized when enforced. Constitutional competences of national authorities to control the execution of the FTA remain therefore unaltered, and if the application of the FTA violates fundamental rights (as the right to health), it can be challenged, which seems not totally compatible with the principle of *pacta sunt servanda* (cf. Lizarazo et al., 2014).

Constitutional case law on FTAs had a strong focus on human rights with particular reference to the right to life and the right to health. This way, Sanitary and Phytosanitary (SPS) measures were upheld because they sought the protection of health in accordance with international treaties (e.g. WTO-SPS measures) and with constitutional regulations (consumer protection, right to a healthy environment, duty of the State to provide public health services, protection of the environment and food production). IPR were linked to the binding commitments concerning public health, biodiversity, traditional knowledge, and the duty to adhere to some pending international conventions besides the international framework whose main parameters are the TRIPS of the WTO, the World Intellectual Property Organization (WIPO) treaties, and the CAN regulations. Therefore, patent registration is subject to these international treaties (Lizarazo et al., 2014).

Although the Court found all the international commitments on IPR rights constitutional, it emphasized the potential and actual impact of IPR on fundamental rights, and therefore, it held that FTAs should be enforced and interpreted according to the Constitutional Bloc,^28^ particularly regarding the right to health, the right to a healthy and diverse environment, and the social goals of the State. Against the arguments in favor of setting aside the US FTA, the Court did not refer to the challenges regarding WTO+ clauses that may affect public health, but in exchange, it held that FTAs can be considered as instruments for the enforcement of the cited rights.^29^ And in any case, the Court maintained that public policies may exclude the patentability of inventions to protect higher constitutional or international interests as health, life of living organisms, and the environment (Lizarazo et al., 2014).

Although the COL-EU FTA has even been more controversial, the Court upheld it.^30^ During the negotiations, this agreement was accused of strengthening IPR with negative consequences for public health and for free competition. Some initial assessments found it stricter than other FTAs, closer to the EU legislation, and without the flexibility accorded in the TRIPS and the Doha Declaration (Ifarma and Fundación Misión Salud, 2009; Seuba, 2009: 65–68).

When revising the COL-EU FTA, the Court held that the parameters of the Doha Declaration are to be applied by the states-parties and that they should respect and implement the Global Strategy and Plan of Action on Public Health, Innovation, and Intellectual Property (GSPA-PHI) of 2008 of the WHO which seeks to avoid IPR abuses but also to avoid restrictive measures to trade and the promotion of technology transfer. When analyzing this FTA, the Court was less explicit in the higher hierarchy of the right to health vis-a-vis IPR. Concerning the regulation of patents, the Court clarified that the adhesion to the Budapest Treaty (Patent Treaty) is a requirement for Peru and Colombia. In addition, the compensation mechanism in case of a ‘non-reasonable reduction’ of the deadline of pharmaceutical patents as a result of the first commercialization of the product was upheld by the Court. Concerning the protection of proof data and other issues related to the security and effectiveness of pharmaceutical products, the Court upheld them based on the argument that these issues have already been analyzed in other international agreements.

Although the final text presented some improvements (i.e. references to the Doha Declaration and to the GSPA-PHI), the Court avoided any discussion concerning patents on pharmaceutical products. As also observed by Seuba and García (2010), the COL-EU FTA is ‘TRIPS-plus’ and the rules of observance seem to be stricter than the ones of the US FTA. In this case, the Court, despite its recognized activism in defense of human rights, avoided any analysis on clauses with potential detrimental effects on public health to Colombia as they seek to give a stricter protection to IPR on pharmaceutical products than the WTO rules, the CAN rules (cf. Decision 689/2008), and even rulings analyzing the COL-US FTA (see also Seuba and García, 2010).

Patents are highly relevant for Colombia because between 1995 and 2010, patent applications increased by almost 52% with a notorious relevance of the pharmaceutical sector and other related sectors of medical devices. It is also the country that has the largest demand for trademarks in the CAN, with pharmaceutical and other medical-related trademarks prominently present (Ramírez, 2012: 31–33). In addition, new constitutional and statutory parameters of the price control of medicines upheld by the Court in 2014 could be considered as a non-accomplishment of FTA IPR clauses. However, the Court omitted to make the link between the FTA’s patent regulations and the constitutional regulation of price control of medicines.

Looking at the US FTA, Colombia has been systematically placed on the Watch List^31^ of the Special 301 Report of the United States. In 2014, some progress in the protection of IPR was recognized, but the role of the Court is starting to be a concern as it affirms that *earlier progress on IPR legislation was reversed in 2013 when the Colombian Constitutional Court invalidated on procedural grounds the law enacting many IPR-related commitments made under the United States-Colombia Trade Promotion Agreement (CTPA)*. Also of concern for the USTA were *Colombia’s limitations on the patentability of certain pharmaceuticals and challenges related to pharmaceutical and agrochemical data protection* (USTR, [2013] 2014: 50). The parameter given by the Court for the price control of medicines and the issue of bio-similar medicines will certainly be an issue for the next report as well as the more flexible analysis of the EU FTA on these issues.

An important issue is how the WIPO, the United States, the EU, and the industry are systematically promoting technical assistance and capacity building on IPR to national authorities (including the judiciary) seeking a better enforcement of FTAs (Abbott, 2007: 12; Ramírez, 2012: 31–33; USTR, [2013] 2014: 46). Although this could be positive, it could also incline the balance in favor of IPR, at the cost of ESCR, which are currently highly protected by some Latin American courts.

## Conclusion

Our hypothesis that the protection of ESCR, and more concretely the right to health, depends also importantly on the will and capacity of national (judicial) actors to give binding character to international and constitutional rules and to address policy failures has been supported by the empirical evidence from the Colombian case, where rights-based judicial adjudication has been crucial. Evidence also suggests that these national actors are only directly supported by the UN, which is highly committed to the universal enforcement of the ICESCR.

The new generation FTAs seek to strengthen the position of IPR holders mainly by an enlargement of the TRIPS and a progressive weakening of the Doha Declaration on Public Health, despite the discourse of adopting it as well as the GSPA-PHI (Roffe and Spennemann, 2006: 85–86; UNDP-UNAIDS, 2012). Although the Court has unanimously upheld all the FTAs, it also promotes the use of constitutional actions against rules that would enforce FTAs when they violate the Constitutional Bloc. This position has been less radical when analyzing the COL-EU FTA. But when potential retaliatory measures against Colombia for not complying with an FTA would be proposed, will human rights rules be considered as an acceptable argument to ignore the FTA commitments?

The Court did not systematically analyze the potential conflict between FTAs and WTO+ or the CAN rules on ESCR issues, in strong contrast with the academic literature and political discourses. Contradictorily, it found that FTAs are constitutional because they respect the WTO and the CAN regulations even if it argued that the constitutional analysis of FTAs does not refer to the compatibility among international rules.

Our article sought to highlight the role of the judiciary (i.e. the Court, and to a lesser extent the ATJ) in the enforcement of ESCR. Although they uphold public policies on trade liberalization and structural adjustments, they enforce ESCR by giving them prevalence over these policies. However, it should be admitted that case law is not the ideal way to regulate public policies on health and that case law of the Court has failures with respect to the technical aspects of trade liberalization and IPR issues. In a way, case law seems also to fill the gaps left by absent health impact assessments (Lee et al., 2007).

So far, regional organizations to which Colombia belongs do not seem to have weighed heavily when it comes to the protection of the right to health. The role of the CAN in balancing free trade and health principles has been marginal, despite the case law of the ATJ. Although the ATJ supported IP administrative agencies in their attempts to balance IPR in favor of access to medicines (Helfer and Alter, 2014), recent developments (TRIPS and the Colombian and Peruvian FTAs) do not seem to lead in that direction. The UNASUR has been trying to go further on these issues, but has still some way to go to arrive at concrete and measurable actions. It remains to be seen whether the remarkable position of the judiciary in the protection of the right to health will be weakened by the proposed training of judges on IPR issues, which may be an attempt to control judicial activism on human rights.

Regional integration policies in Latin America should try to fill gaps in the fields where the member states independently lack capacities, such as the improvement and coverage of health care in border regions, but also with respect to innovation chains seeking technological progress in pharmaceutical and treatment areas in order to be less dependent on multinationals and to reach reasonable prices of medicines not only by means of price controls and patent limits.

European and US human rights conditionality in FTAs refers mostly to first-generation rights, not to ESCR, promoting clauses with potential negative effects of certain IPR-related clauses on the enjoyment of the ESCR by the trading partners’ populations. New generation FTAs, mainly promoted by these economic blocks, tend to restrict the flexibility of the TRIPS and the related Doha Declaration, but they can also be seen as a disintegrating factor as in the case of CAN by eliminating regional rules that limited IPR to the benefit of social policies of the member states and as in the case of the ongoing competition between integration models (Pacific Alliance *vs* UNASUR).

A final – more general – conclusion refers to the implications of our analysis for the emerging field of global and regional health policy and health governance studies. This literature is convincing in its normative theorizing, but its empirical analyses might sometimes suffer from selection biases. The observed global or regional policy developments that corroborate normative positions in favor of more policy intervention at those levels are not always very deep and are sometimes (over-)compensated by global or regional health-related developments in other policy areas (e.g. trade) or within overlapping regional arrangements.

## Notes

1. For a good overview of this literature, see Riggirozzi (2015).

2. See ICJ (2008) and Forman et al. (2013).

3. Statutory Law 1751/15 (Constitutional right to health), upheld by the Court Ruling 313/14.

4. Comisión Nacional de Precios de Medicamentos (CNPM): Circular 04/2006.

5. In addition, it was estimated that Trade-Related Aspects of Intellectual Property Rights (TRIPS)-plus provisions in the US-Colombia free trade agreement (FTA) would heavily increase expenditure on medicines by 2020, possibly provoking a drop in medicine consumption by 40% (Gamba, 2006; quoted by UNDP-UNAIDS, 2012: 4).

6. Conpes Social Document 155/12

7. Auto 263/12

8. Cf. Circulares 1-2/2014 from the Comisión Nacional de Precios de Medicamentos y Dispositivos Médicos.

9. Ruling 313/14

10. Bio-generics are used in the European Union (EU), but also in other Latin American countries as Brazil, Argentina, and Mexico (Feijó et al., 2012, 2014; Minsalud, 2014).

11. http://www.minsalud.gov.co/Politicas%20Farmaceuticas/Biotecnol%C3%B3gicos/Comentarios%20recibidos%20biotecnologicos%20-%201%20ronda/BIO%20Comments%20on%20Colombia%20Biologics%20Regulations%20FINAL.pdf

12. See also, European Commission, Enterprise and Industry Directorate General ref: Ares (2013) 3206827-08/10/13

13. http://www.who.int/medicines/areas/policy/doha_declaration/en/

14. The annual global review of Intellectual Property Rights (IPR) protection and enforcement by the Office of the US Trade Representative (USTR).

15. UNDP-UNAIDS (2012) identified the main TRIPS-plus provisions that potentially threaten the access to medicines: (1) *Broadening Patentability* or *the evergreening of pharmaceutical patents*, that is, the registration of new forms and new uses of known substances to extend the patent by modifying the molecules; (2) *Restricting Patent Oppositions*, seeking to avoid *patent applications that do not fulfill the requirements in national legislation for granting the patents*; (3) *Extending Patent Duration beyond the 20 years* at the cost of the use of generics; (4) *Introducing Test Data Exclusivity and a Patent-Registration Linkage*; and (5) *IP Enforcement Requirements*, for example, by seizing essential medicines in transit.

16. Cf. Annex II to the Additional Protocol to the Frame Agreement of the Pacific Alliance, IV sector (social services).

17. *Organismo Andino de Salud Convenio Hipólito Unanue.* The health ministries of Bolivia, Colombia, Chile, Ecuador, Peru, and Venezuela participated.

18. Amazonian Cooperation Treaty Organization, whose member states are Bolivia, Ecuador, Colombia, Peru, Brazil, Venezuela, Guyana, and Surinam.

19. The mains issues treated were the Andean epidemiological profile, universal access to medicines, the management of human resources in health, and the universalization of health systems.

20. See, for example, Riggirozzi (2014, 2015).

21. It establishes the common regime on industrial property and its adaptation to the compromises adopted in the TRIPS agreement of the World Trade Organization (WTO).

22. In 2013, the Andean Tribunal of Justice (ATJ) upheld Decision 689 because it respects the common regime of the Andean Community (CAN) (Decision 486). It is a ‘Decision 486+’ scheme because it reflects the minimum criteria that may be complemented by national legislations of member states (ref 01-AN-2010).

23. Decision 689 mainly allows the following rules that are of relevance for the health sector: (1) to extend the deadline to vindicate the right to priority up to 2 months; (2) to request additional specifications in the description of the invention; (3) except in the case of the pharmaceutical patents, to create ways to compensate the owner of a patent for unreasonable delays of national authorities in the registration of the patent; (4) the establishment of the *bolar clause*, that is, member states may authorize the use of protected materials by a patent to generate the needed information to support the application to commercialize a product; (5) the establishment of a multiclass register of trademarks and a regime of border measures for trademarks of products in transit.

24. For example, recently the CAN ordered the Colombian Superintendency of Trade and Industry to modify an administrative instruction (Res 21447/12) to concede patents, which was signaled as an important advance in the promotion of IPR (Ramírez, 2012: 31). The instruction allowed to patent therapeutic procedures, which is forbidden by the CAN (cf. http://www.portafolio.co/economia/can-ordena-colombia-reversar-instructivo-patentes).

25. Study on Specialized Intellectual Property Courts (IIPI – US Patent and Trademark Office, 2012), quoted by Ramírez (2012: 31–33).

26. For example, Médecins sans frontières (2004), Zerda, Cortés, De la Hoz, Durán and Sarmiento (2005), Malpani and Bloemen (2009), Nasu (2010), and Garay et al. (2011: 137–165).

27. See Lizarazo et al. (2014).

28. It is formed by the main international treaties protecting human rights and the International Humanitarian Law together with the Colombian Constitution.

29. The Court argued as follows: (1) the parties may adopt measures to protect public health by promoting universal access to medicines, according to the Doha Declaration on TRIPS and public health; (2) the mechanisms to avoid unjustified delays in the recognition of patents as compensatory measures for non-pharmaceutical products seek to comply with the constitutional principle of administrative efficiency; (3) the application of TRIPS mechanisms of flexibility in case of national (health) emergency (by granting compulsory licenses and import medicines without the authorization of the holder of the patent) is a way of balancing the general interest with the social obligations of IPR; and (4) Colombia keeps the competence to decide the way to fulfill these duties, and in any case the right to health has a higher hierarchy than IPR.

30. C 335/14.

31. The USTR ([2013] 2014) defined ‘Priority Watch List’ and ‘Watch List’ as categories of trading partners with specific concerns regarding *IPR protection, enforcement, or market access for persons relying on IPR*’. The Priority Watch List receives an ‘*increased bilateral attention concerning the problem areas* (p. 58).
